# Antibiotic Susceptibility profile of *Staphylococcus aureus* isolated from sausages in Meknes, Morocco

**DOI:** 10.14202/vetworld.2018.1459-1465

**Published:** 2018-10-19

**Authors:** Abdelaziz Ed-Dra, Fouzia Rhazi Filali, Aziz Bouymajane, Faouzia Benhallam, Abdellah El Allaoui, Abdellah Chaiba, Filippo Giarratana

**Affiliations:** 1 Team of Microbiology and Health, Laboratory of Chemistry-Biology Applied to the Environment, Moulay Ismail University Faculty of Science, BP. 11201 Zitoune Meknes, Morocco; 2 SADS–CRMEF Souss Massa Daraa Inezgane, Agadir, Morocco; 3 Department of Veterinary Science, University of Messina, Polo Universitario della Annunziata, 98168 Messina, Italy

**Keywords:** antimicrobial resistance, foodborne disease, infection, sausages, *Staphylococcus aureus*

## Abstract

**Background and Aims:**

*Staphylococcus aureus* is one of the most common causes of foodborne disease worldwide, due to the consumption of food contaminated by their toxins. This study aimed to determine the prevalence and the antimicrobial resistance of *S. aureus* isolated from sausages in Meknes city of Morocco.

**Materials and Methods:**

A total of 156 samples (Beef sausages, Turkey sausages, and Artisanal sausages “Merguez”) were collected from different shopping sites (butchery, supermarket, street vendors, and weekly market “Souk”) and used for the isolation of *S. aureus*. All the isolated strains were tested for their antimicrobials resistance to 16 antibiotics.

**Results:**

Our results showed the presence of *S. aureus* in 63 samples (40.38%). Furthermore, the antimicrobial resistance study showed that 84.13% of isolated *S. aureus* were resistant to streptomycin, 76.20% to tetracycline, 42.86% to ampicillin, 41.27% to doxycycline, 38.1% to penicillin G, and 19.05% to chloramphenicol with the presence of 25 different phenotypic profiles. However, all isolated strains were sensitive to oxacillin, cefoxitin, gentamicin, and vancomycin.

**Conclusion:**

The findings of this study revealed consumption of sausages as a potential risk of foodborne poisonings because of its contamination with the multi-resistant strains of *S. aureus*. Moreover, this contamination is related to the season, sampling sites and the origin of the raw material.

## Introduction

Foodborne diseases are a major public health concern worldwide and are defined as a disease of infectious or toxic nature caused by, or thought to be caused by, the consumption of contaminated food or water [1]. Staphylococcal foodborne disease is one of the most frequent universal foodborne diseases, and it is caused by the ingestion of food contaminated with enterotoxins produced by some strains of *Staphylococcus* [2,3]. Its symptoms have a rapid onset (2-6 h) and may include vomiting, stomach pain, and diarrhea [4].

The genus *Staphylococcus* is placed in the family Micrococcaceae. This genus is divided into coagulase-positive staphylococci and coagulase-negative staphylococci based on their ability to coagulate plasma [5]. *Staphylococcus aureus* is the most significant human pathogen among the staphylococci. It is a ubiquitous spherical bacterium, Gram-positive and facultative anaerobic. He can grow in a wide range of pH (between 4.2 and 9.3), temperatures (between 7°C and 48.5°C), and in a high concentration of sodium chloride (15%) [1]. These characteristics favor the growth of this bacterium in many food products. Indeed, previous studies have isolated *S. aureus* from various food of animal origin [6-12].

Worldwide, *S. aureus* is considered the third common pathogen that causes food poisoning [13]. In the United States, *S. aureus* is considered one of the top five pathogens causing domestically acquired foodborne diseases and is responsible for an estimate of 241,000 illnesses per year [10,14]. In 2009, 23 cases of food poisoning were reported in France due to the consumption of raw milk and cheese contaminated with *S. aureus* [15]. A study in Morocco showed that *S. aureus* was responsible for 72% of foodborne outbreaks [16]. Moreover, the delegation of epidemiology and disease control reported 13,339 cases of foodborne diseases between 2001 and 2010 of which 31% were caused by *S. aureus* [17].

The extended use and misuse of antibiotics in agriculture, stock farming, veterinary medicine, and treatment of human diseases increase the resistance of bacteria to antimicrobial agents. In livestock farms, different antimicrobial agents are used extensively in sub-therapeutic/therapeutic doses for growth promotion, routine disease prevention, and treatment of bacterial diseases [18,19]. This indiscriminate practice is usually worse in developing countries including Morocco, where there are no strict controls on the use of antimicrobials in food-producing animals [20]. This has led to increased resistance to different antimicrobials used in these fields [11,21-23]. In the last years, *S. aureus* resistant to methicillin (MRSA) and vancomycin (VRSA) was isolated from different samples [1,7,8,10,21,23-26], these antibiotics are a choice drug for the treatment of cases infected by this bacterium; hence, the World Health Organization has triggered the alarm signal about these resistances and ranked the MRSA and VRSA among the high priority for searching the new and effective antibiotic treatments [27].

In this context, this study aimed to evaluate the prevalence of *S. aureus* in sausages sold in Meknes city (Morocco) and to determine the antimicrobial susceptibility of isolated strains.

## Materials and Methods

### Ethical approval

Ethical approval was not required in this study since no live animals were used in the experiments.

### Samples collection and microbiological analysis

A total of 156 samples of sausages distributed as follow: 60 of turkey sausages, 60 of beef sausages, and 36 of “Merguez” sausages were randomly collected from various local supermarkets, weekly market, butcheries, and street vendors. The collection was carried out during 1 year from March 2014 to February 2015. The samples were aseptically collected, and each sample was placed in a separate, sterile plastic bag. The samples were brought under refrigeration to the laboratory and analyzed within the following 2 h. The samples (25 g) were weighed into sterile stomacher bags diluted with 225 mL sterile buffered peptone water (Biokar) and homogenized in a stomacher for about 1 min; 0.1 mL was streaked on Baird-Parker (BP) agar (Biokar) supplemented with egg yolk tellurite emulsion and incubated at 37°C for 24-48 h. Strains cultured on BP agar medium were identified as *S. aureus* if growth was observed and the colonies showed the typical morphologic characteristics (black colonies with an opaque precipitation halo). The tube coagulate test was determined and evaluated for coagulation after 3 and 24 h of incubation.

### Antibiotic susceptibility

The resistance pattern of *S. aureus* was determined using the disk-diffusion test [28]. 16 antibiotics (Oxoid) were chosen for the study based on the most used active principles in human medicine, national veterinary therapy, and according to their common use in research. The use of oxacillin/methicillin is not usually used in veterinary practice, and it was included in this study only for epidemiological purposes; the drugs tested are indicated in Table-1. Multiple antibiotic resistance (MAR) index is calculated as the ratio of some resistance antibiotics to the total number of antibiotics to which the isolates are exposed. *S. aureus* ATCC 29213 was used as a control strain.

**Table-1:** Antimicrobial agents and the range of concentrations tested.

Antimicrobial agents	Code	Concentration disc (μg)
Penicillin G	P	6
Ampicillin	AMP	10
Oxacillin	OX	5
Cefoxitin	FOX	30
Gentamicin	GN	30
Kanamycin	K	30
Fusidic acid	FD	10
Ofloxacin	OFX	5
Enrofloxacin	ENR	5
Erythromycin	E	15
Tetracycline	TE	30
Doxycycline	DXT	30
Vancomycin	VA	30
Chloramphenicol	C	30
Streptomycin	S	10
Trimethoprimsulfamethoxazole	SXT	1.25/23.75

### Statistical analysis

The data were presented as means±standard error, and the statistical analyses were performed using Microsoft Office Excel (2010). The comparison of contamination averages was performed using the student test with p<0.05.

## Results

### Contamination rate of sausages by staphylococci

**Figure-1:**
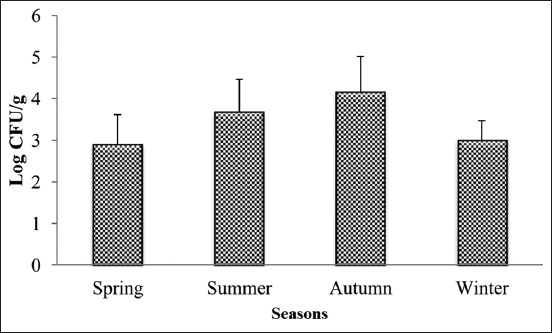
The average values of staphylococci counted in sausages according to seasons sampling.

**Figure-2:**
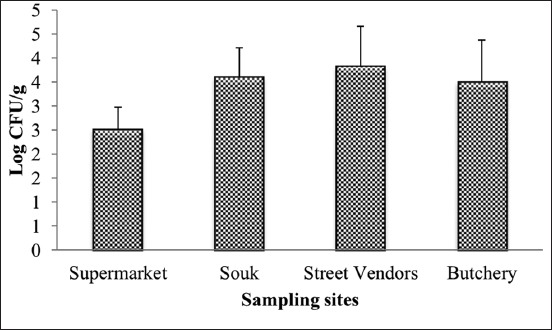
The average values of staphylococci counted in sausages according to sampling sites.

**Figure-3:**
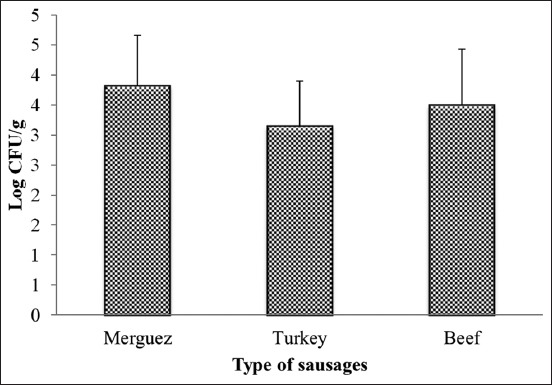
The average values of staphylococci counted in sausages according to sausages types.

This study was carried out during 1 year to evaluate the contamination level of sausages by staphylococci, and to study the effect of the different factors influencing this contamination. The results of this study show that the average rate of contamination with staphylococci was 3.42±0.88 log cfu/g with a minimum value of 1.47 log cfu/g and a maximum value of 5.6 log cfu/g. The study of seasonal effect shows that staphylococci take a maximum value during autumn (4.15±0.86 log cfu/g), followed by summer (3.68±0.78 log cfu/g), winter (3.00±0.46 log cfu/g), and spring (2.90±0.71 log cfu/g) (Figure-1), with a significant difference between the cold seasons (winter and spring) and the hot seasons (autumn and summer) (p<0.05). Furthermore, the study of the sampling sites effect shows that sausages sold at street vendors are the most contaminated with staphylococci (3.82±0.84 log cfu/g), followed by souk (3.6±0.61 log cfu/g), butchery (3.5±0.88 log cfu/g), and the supermarket (2.51±0.46 log cfu/g) (Figure-2), while the sausages sold in supermarket differ significantly from those sold in the others sites (p<0.05). In the other hand, the result of this study showed that the raw material origin has a significant effect on the contamination rate of sausages by staphylococci (p<0.05); the artisanal sausages “Merguez” is the most contaminated (3.82±0.84 log cfu/g), followed by beef sausages (3.5±0.93 log cfu/g) and turkey sausages (3.15±0.75 log cfu/g) (Figure-3).

### Prevalence of S. aureus in sausage samples

The isolation and identification of *S. aureus* were performed according to the method described previously. From 156 analyzed samples, 63 were positive for *S. aureus* (40.38%). These results showed that *S. aureus* governs the summer season (Table-2); furthermore, the study of sampling sites effect showed that the sausages sold in street vendors were the most contaminated with 50% (Table-3). On the other hand, the artisanal sausages “Merguez” was the most contaminated with *S. aureus* according to the raw material origin (Table-4).

**Table-2:** Effect of sampling seasons on sausages contamination with *S. aureus*.

Sampling seasons	Number of samples		

	Analyzes	Positives	Percentage
Spring	39	16	41.02
Summer	39	29	74.36
Autumn	39	14	35.89
Winter	39	4	10.25

*S. aureus=Staphylococcus aureus*.

**Table-3:** Effect of sampling sites on sausages contamination with *S. aureus*.

Sampling sites	Number of samples		

	Analyzes	Positives	Percentage
Butchery	72	28	38.88
Supermarket	24	3	12.5
Street vendors	36	18	50
Weekly market	24	14	58.33

*S. aureus=Staphylococcus aureus*.

**Table-4:** Effect of raw material origin on sausages contamination with *S. aureus*.

Origin	Number of samples		

	Analyzes	Positives	Percentage
Turkey sausage	60	22	36.66
Beef sausage	60	23	38.33
Artisanal sausages “Merguez”	36	18	50

*S. aureus=Staphylococcus aureus*.

### Antibiotic susceptibility test

The antibiotic susceptibly was carried out according to the method described by the Clinical Laboratory Standard Institute. The isolated *S. aureus* (63) was tested for 16 antibiotics belonging to different groups, and the results are presented in Table-5. This results showed that 61 isolates (96.82%) were resistant to at least one antibiotic, 56 (88.88%) were resistant to two or more antibiotics, and 44 (69.84%) were resistant to three or more antibiotics (considered as Multi-resistant). Moreover, the analysis of our results showed that the MAR index varies between 0 and 0.56 with the presence of 25 different phenotypic profiles (Table-6).

**Table-5:** Antimicrobial resistance percentages of isolated *S. aureus*.

Antibiotics	Number of S. aureus isolates (n=63)		

		S (%)	R (%)
Penicillin G (6 μg)	P	39 (61.9)	24 (38.1)
Ampicillin (10 μg)	AMP	36 (57.14)	27 (42.86)
Oxacillin (5 μg)	OX	63 (100)	0 (0)
Cefoxitin (30 μg)	FOX	63 (100)	0 (0)
Gentamicin (30 μg)	CN	63 (100)	0 (0)
Kanamycin (30 μg)	K	59 (93.65)	4 (6.35)
Fusidic acid (10 μg)	FD	62 (98.41)	1 (1.59)
Ofloxacin (5 μg)	OFX	54 (85.71)	9 (14.29)
Enrofloxacin (5 μg)	ENR	54 (85.71)	9 (14.29)
Erythromycin (15 μg)	E	60 (95.24)	3 (4.76)
Tetracycline (30 μg)	TE	15 (23.80)	48 (76.20)
Doxycycline (30 μg)	DXT	37 (58.73)	26 (41.27)
Vancomycin (30 μg)	VA	63 (100)	0 (0)
Chloramphenicol (30 μg)	C	51 (80.95)	12 (19.05)
Streptomycin (10 μg)	S	10 (15.87)	53 (84.13)
Trimethoprimsulfamethoxazole (1.25 μg/23.75 μg)	SXT	61 (96.82)	2 (3.18)

**Table-6:** Resistance profile of isolated *S. aureus*.

Number of antibiotics	Resistance profile	Number of Isolates	MAR
0		2	0
1	TE	4	0.06
	S	1	0.06
2	S, TE	9	0.125
	TE, C	1	0.125
	OFX, ENR	1	0.125
	S, AMP	1	0.125
3	S, AMP, P	9	0.19
	S, TE, DXT	6	0.19
	S, TE, C	2	0.19
	S, TE, SXT	1	0.19
4	S, TE, DXT, C	4	0.25
	S, TE, DXT, SXT	1	0.25
	S, TE, OFX, ENR	1	0.25
5	S, TE, DXT, AMP, P	7	0.31
	S, TE, DXT, C, P	2	0.31
	S, AMP, OFX, ENR, K	1	0.31
	TE, DXT, AMP, P, E	1	0.31
	TE, DXT, OFX, ENR, E	1	0.31
	S, TE, DXT, C, FD	1	0.31
	S, TE, DXT, AMP, C	1	0.31
6	S, TE, AMP, P, ENR, OFX	2	0.37
	S, TE, DXT, AMP, P, K	1	0.37
7	S, TE, AMP, P, OFX, ENR, K	2	0.43
9	S, TE, DXT, AMP, P, C, OFX, ENR, E	1	0.56

Among the tested antibiotics, the isolated strains were resistant to streptomycin (84.13%), followed by tetracycline (76.20%), ampicillin (42.86%), doxycycline (41.27%), and penicillin G (38.1%). On the other hand, they were sensitive to oxacillin, cefoxitin, gentamicin, and vancomycin (Table-5).

## Discussion

The genus *Staphylococcus* includes at least 40 species which are pathogenic bacteria causing a broad spectrum of diseases with varying degrees of severity; their natural habitat includes humans, animals, and environment. The presence of staphylococci in food is considered a major risk to the public health, for this reason, its limit of acceptability in sausages was fixed at 5.10^3^ cfu/g in Morocco.

The results of our study showed that the contamination rate of sausages with staphylococci has an average rate of 3.42±0.88 log cfu/g with a minimum of 1.47 log cfu/g and a maximum of 5.6 log cfu/g, of which 31.41% exceeds the limit and are unfit for consumption. These results are similar to those found in Turkey [29], lower than those reported in Nigeria [30], and higher than the results carried out in Jordan [31]. However, a study carried out previously in Rabat city (Morocco) showed that the contamination of poultry meat with staphylococci has an average rate of 2.67 log cfu/g [32].

Our finding showed the presence of *S. aureus* in 63 samples (40.38%). These results are similar to that found in the imported meats in South Korea (40.94%) [15] and fresh sausages samples in Egypt (45%) [33]. They are higher than that reported in poultry meat in Rabat city of Morocco (16.66%) [32], chicken samples in China (24.2%) [23], analyzed meat samples in Italy (10%) [34], and fresh meat samples in Shanghai (28.1%) [35]. However, they are lower than that reported in turkey samples in the USA (64.2%) [10]. In Italy, the results published by Pesavento *et al*. [6] showed the presence of *S. aureus* in 23.86% of analyzed samples, with 28.57% in poultry meat, 29.41% in beef meat, and 15.15% in pork meat. The high contamination of sausages with *S. aureus* may occur directly from contaminated raw material or may results from poor hygiene during production processes or at the retail and storage stage [36].

The massive use of antibiotics in feed to promote growth and the inappropriate use of antimicrobials agents in veterinary and human medicine are considered to be major contributors to the emergences of resistance [20,37]. Moreover, *S. aureus* is notorious for its ability to become resistant to antimicrobials due to their capacity to produce an exopolysaccharide barrier and because of their location within microabscesses, which limit the action of drugs [38]. In the other hand, the acquisition of resistance genes by horizontal transfer has high importance; some studies prove the presence of different genes such as *tetM*, *mecA*, and *blaZ* that are responsible for the resistance to tetracycline, oxacillin, and penicillin, respectively [39].

The results of our study showed that 96.82% of isolated *S. aureus* were resistant to at least one antibiotic, 88.88% were resistant to two or more antibiotics, and 69.84% were resistant to three or more antibiotics. A study in Italy showed that 68.8% of analyzed *S. aureus* were resistant to at least one antibiotic [34]. In Jordan, about 88.5% of the *S. aureus* exhibited resistance to at least one antibiotic in imported fresh fish samples [40]. In the United States, 52% of the *S. aureus* isolated from meat and poultry samples were multi-resistant [19].

The antimicrobial analysis showed that 84.13% of isolated *S. aureus* are resistant to streptomycin, this result is higher than that found in retail chicken in Egypt (52.1%) [41], and in food samples in Iran (5.8%) [39]. Furthermore, 76.20% of isolated strains were resistant to tetracycline; this result is similar to that found in retail chicken in Egypt taking in consideration the intermediate and resistance profiles [41], but higher than that found in Italy (19.04%) [6], Jordan (36.5%) [39], and Iran (29.6%) [42].

The rate of resistance to ampicillin and penicillin G was 42.86% and 38.1%, respectively. The resistance to these antibiotics is common in *S. aureus* and has been observed previously in Malaysia (72.30% ampicillin and 53.38% penicillin) [43], Iran (77.3% ampicillin and 76% penicillin G) [44], and Italy (42.86% ampicillin and 16.66% penicillin G) [6]. On the other hand, the isolated strains of *S. aureus* were sensitive to gentamicin, oxacillin, cefoxitin, and vancomycin.

The high percentage of resistant *S. aureus* isolates to these antibiotics could be due to the widespread administration of these antimicrobials to control and treat infections on dairy farms [39]. Furthermore, MAR index analysis showed the presence of 25 different phenotypic profiles among the 63 strains. This diversity can be explained by the different sources of contamination of sausages since the preparation of the raw material (slaughter, evisceration, cutting) until the manufacturing, storage, and sales in different sites [36,45].

## Conclusion

The high level of contamination of sausages with *S. aureus* highlights the poor hygiene all along the chain of manufacture and sale of this product. Furthermore, our study showed that the consumption of this product might be a potential risk of foodborne infection. Fortunately, we have not found strains resistant to methicillin and vancomycin, but this resistance can be acquired from the medical or veterinary fields to the food chain product. Hence, it is interesting to survey the resistance profile of this bacterium in all the stage manufacturing process and applied the good practices of hygiene and Hazard Analysis Critical Control Point especially in informal sales sites.

## Authors’ Contributions

This work was carried out in collaboration between all authors. AE, FRF, and FB designed the experimental procedures. AE, AB, AEA, and AC conducted the experimental analysis. AE, FRF, and FG analyzed the data and wrote the manuscript. All authors read and approved the final manuscript.
